# Case Report: Plasticity in Central Sensory Finger Representation and Touch Perception After Microsurgical Reconstruction of Infraclavicular Brachial Plexus Injury

**DOI:** 10.3389/fnins.2022.793036

**Published:** 2022-02-25

**Authors:** Jennifer Ernst, Thomas Weiss, Nadine Wanke, Jens Frahm, Gunther Felmerer, Dario Farina, Arndt F. Schilling, Meike A. Wilke

**Affiliations:** ^1^Department of Trauma Surgery, Orthopaedics, and Plastic Surgery, Universitätsmedizin Göttingen, Göttingen, Germany; ^2^Clinical Psychology, Friedrich Schiller University Jena, Jena, Germany; ^3^Fakultät Life Sciences, Hamburg University of Applied Sciences (HAW Hamburg), Hamburg, Germany; ^4^Biomedizinische Nuclear Magnetic Resonance (NMR) Forschungs-GmbH am Max-Planck-Institut für biophysikalische Chemie, Göttingen, Germany; ^5^Department of Bioengineering, Imperial College London, London, United Kingdom

**Keywords:** brachial plexus injury, brain plasticity, somatosensory, S1, sensory cortex

## Abstract

After brachial plexus injury (BPI), early microsurgery aims at facilitating reconnection of the severed peripheral nerves with their orphan muscles and sensory receptors and thereby reestablishing communication with the brain. In order to investigate this sensory recovery, here we combined functional magnetic resonance imaging (fMRI) and tactile psychophysics in a patient who suffered a sharp, incomplete amputation of the dominant hand at the axilla level. To determine somatosensory detection and discomfort thresholds as well as sensory accuracy for fingers of both the intact and affected hand, we used electrotactile stimulation in the framework of a mislocalization test. Additionally, tactile stimulation was performed in the MRI scanner in order to determine the cortical organization of the possibly affected primary somatosensory cortex. The patient was able to detect electrotactile stimulation in 4 of the 5 fingertips (D1, D2, D4, D5), and in the middle phalanx in D3 indicating some innervation. The detection and discomfort threshold were considerably higher at the affected side than at the intact side, with higher detection and discomfort thresholds for the affected side. The discrimination accuracy was rather low at the affected side, with stimulation of D1/D2/D3/D4/D5 eliciting most commonly a sensation at D4/D1/D3/D2/D5, respectively. The neuroimaging data showed a mediolateral succession from D2 to D5 to D1 to D4 (no activation was observed for D3). These results indicate a successful regrowth of the peripheral nerve fibers from the axilla to four fingertips. The data suggest that some of the fibers have switched location in the process and there is a beginning of cortical reorganization in the primary somatosensory cortex, possibly resulting from a re-education of the brain due to conflicting information (touch vs. vision).

## Introduction

Brachial plexus injury (BPI) is a complex injury, mostly occurring in young men after motorbike accidents, typically resulting in lifelong motor and sensory dysfunctions and neuropathic pain of varying severity (Midha, 1997; Giuffre et al., 2010). Recovery is usually slow due to the physiological regrowth velocity of the axons (Waller, 1850). Injured peripheral nerve cells can regenerate their axons if given an appropriate guide. Consequently, the state-of-the-art treatment is nerve autograft between the two nerve ends (Narakas, 1985; Nagano, 1998; Millesi, 2001). Early, appropriate reconstruction of the nerve continuity allows for directed axonal regrowth and procedures can prevent irreversible atrophy of the denervated muscles (Belzberg et al., 2004). This usually leads to preservation of elbow function (Nagano et al., 1989), while the functional restoration of the distal hand muscles remains poor, especially for the lumbrical muscles (Sakellariou et al., 2014).

The aim of the present case study was thus to investigate sensory recovery in a microsurgically reconstructed BPI patient 2 years after injury. To this end, we combined electrotactile stimulation and sensory mapping in the fMRI to explore the central sensory finger representation of the patient's affected side.

## Case Description

The study was approved by the local ethics committee (no. 11-10-14, version 6.1) and signed informed consent was given by the subject.

**Figure 1:**
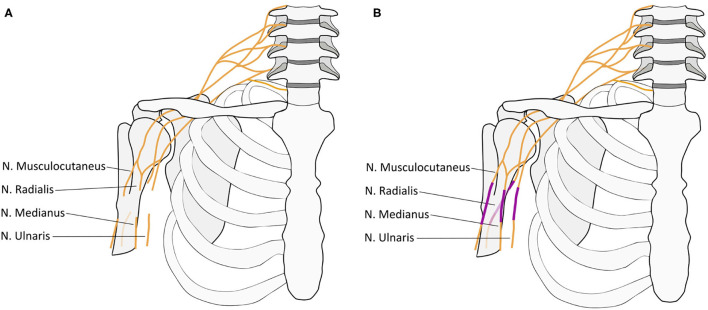
Visualization of nerve injury and reconstruction. **(A)** The interrupted nerves indicate the level of disruption of the infraclavicular brachial plexus distal to the conjunction of the ventral roots of C5-7 (lateral cord) and C8 and T1 (medial cord) to the final contributions of the median nerve. The proximal and distal nerve ends were retracted toward the forearm by up to 7 cm. From the union of the lateral and medial root to the median nerve the proximal ends of the radial, ulnar, median, and musculocutaneous nerves could be identified. **(B)** Sural nerve and median cutaneous antebrachii nerve grafts (indicated in violet) were doubled, reversed, and used to bridge the gaps between the proximal and distal nerve stumps by epineural suture under loupe magnification.

A 20-year old man suffered a sharp subtotal amputation of his right, dominant arm at the level of the axilla, falling through a French door. After emergency reconstruction of the axillary artery within <6 h by an autologous transplant he developed a compartment syndrome following reperfusion on day two after the revascularization what was treated by immediate fasciotomy. A second look revealed viable muscles at the upper-/forearm and hand. Six days after the injury, the nerve was microsurgically reconstructed, using a combined infra- and supraclavicular surgical approach. The disruption of the nerves was found infraclavicular with a distance from the injury to the hand of approximately 110 cm. The distal nerve ends were retracted toward the forearm by up to 7 cm. From the union of the lateral and medial root of the median nerve upward, the proximal ends of the radial, ulnar, median, and musculocutaneous nerves could be identified. Sural nerve and median cutaneous antebrachii nerve grafts were doubled, reversed, and used to bridge the gaps between the proximal and distal nerve stumps by epineural suture under loupe magnification (Figure 1). Consistent with a nerve regeneration rate of 1–3 mm/day (Seddon et al., 1943), 22 months later full motor recovery of the elbow and wrist was observed. Clinical, neurophysiological sEMG follow-up measurements showed voluntary contraction and ongoing reinnervation of the reinnervated upper- and (fore-)arm muscles (flexion/extension; supination/pronation) and single action potentials of the extensor carpi ulnaris muscle. For the interosseus and abductor pollicis muscle, it was not possible to measure voluntary action potentials at the end of the observation period, which corresponds to the clinically manifest hand paralysis with no active movement and claw deformity. With this physical limitation, the patient described that he was able to continue with his life as before. A psychological screening after the accident showed no evidence of depression, anxiety, or post-traumatic stress disorder. No adverse and unanticipated events occurred during the observation time.

## Timeline

The microsurgery was performed 6 days after the injury and the electrotactile and fMRI measurements were collected 22 months after nerve injury (Supplementary Figure 1).

## Diagnostic Assessment

### Methods

#### Psychophysics 1: Electrotactile Stimulation

Tactile psychophysics were used in combination with fMRI to study the sensory input of the fingers both at a perceptual level and in the primary sensory cortex (S1).

**Figure 2:**
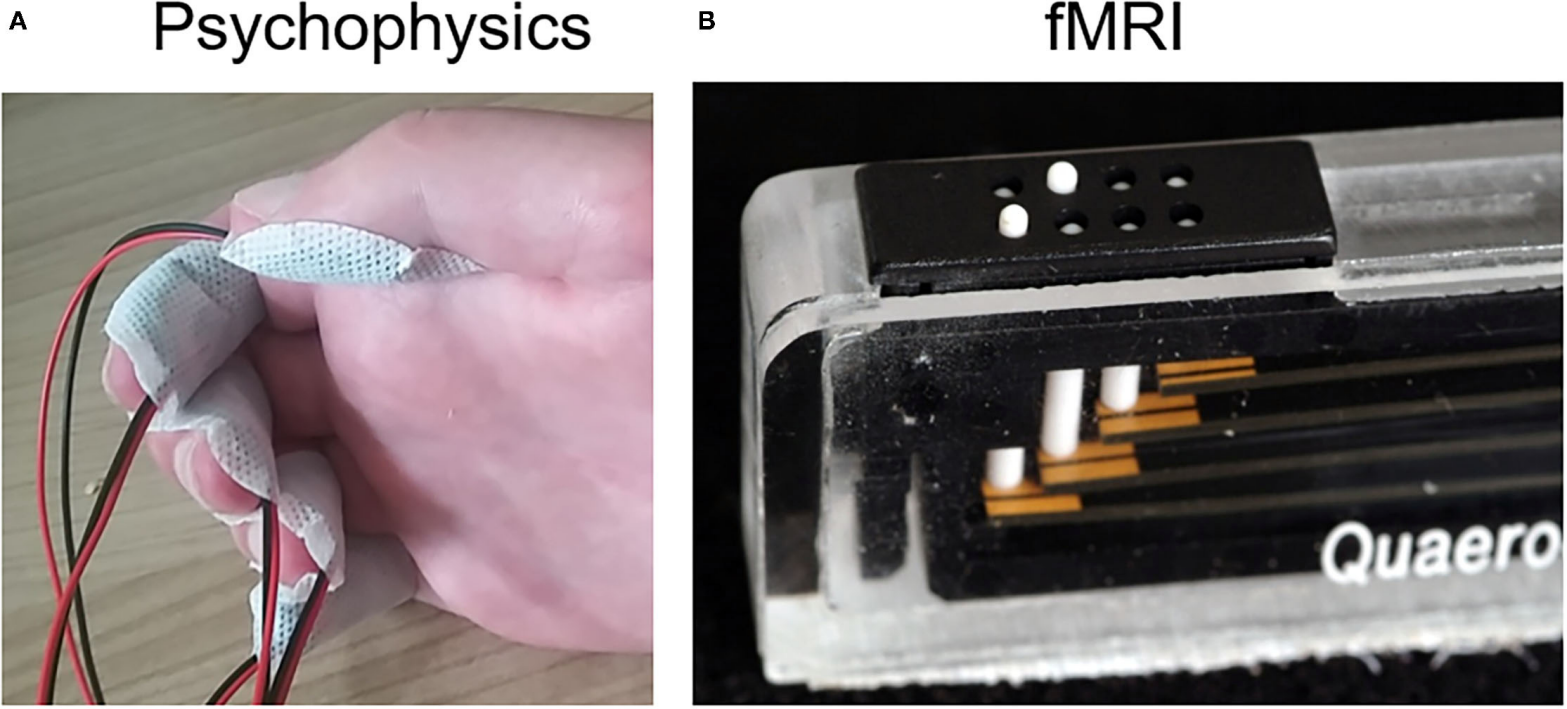
Stimulation types. **(A)** Electrotactile stimulation. Five electrodes were fixed on the according fingertips for psychophysical examination. **(B)** Tactile stimulation. Five piezo-electric vibrators were used for somatosensory stimulation during fMRI, one at each finger, each with an 8-dot Braille display.

The subject sat in front of a desk, with the hand resting on a cushion. First, the affected, then the unaffected hand was stimulated. The protocol was identical for both hands. It comprised of the multichannel stimulator RehaStim 1 (Hasomed, Magdeburg, DE) connected to five self-adhesive concentric electrodes (CoDe1.0, 4-cm diameter, Spes Medica, IT) used for electrotactile stimulation (Figure 2A). The stimulator was controlled via a custom-made graphical user interface (GUI) programmed in MATLAB (MathWorks, USA). The electrotactile bipodal stimulation was run at a frequency of 32 Hz, with varying intensity (see below). The stimulation electrodes were placed on the subject's five fingertips, covering the distal phalanx of each finger (Figure 2A). Only for the middle finger, the electrode was placed slightly lower (covering also the middle phalanx), because no sensation was perceived at the distal phalanx even at 10 mA pulse intensity.

Stimulation parameters were selected in accordance with previous studies (Hashimoto et al., 1999a,b; Blankenburg et al., 2003; Schweisfurth et al., 2016). As reported before, these parameters were suitable to identify threshold values for the unaffected side. For the affected side, the same parameters were applied, with the exception that the upper boundary for pulse width was set to 500 μs in order to avoid harm to the affected side.

##### Task

First, stimulation intensity and pulse width were adjusted for each electrode to measure sensation thresholds as well as to determine a clearly detectable sensation without discomfort. For each channel of the affected hand, the pulse intensity was originally set to 2 mA. At this intensity, the pulse width was increased in steps of 5 μs every 2 s until the subject reported a sensation for the first time. If no sensation was reported until 250 μs, the pulse intensity was increased by 2 mA and the protocol was repeated until the subject reported a sensation. Once the subject reported a sensation (PW_sen1_), the stimulation was interrupted, and the sensation threshold was determined a second time (PW_sen2_) at the same pulse intensity (PI). Next, the stimulation was further increased until the subject first reported a very defined and clear sensation (PW_def_) and then a slight discomfort (PW_unc_). At this PI, PW_sen1_, PW_sen2_, PW_def_, and PW_unc_ were determined for that finger at the intact hand. After PI/PW_def_ values were determined for each finger, the subject was asked whether they were perceived at similar intensity across the five fingers. The PW_def_ values were adjusted until similar perceptions were achieved. Then, the subject took part in a mislocalization test, as adapated from (Schweizer et al., 2000). He received stimulation (with the respective PI and PW_def_) at random locations within a hand (first the affected, then the intact) and was asked to report at which finger he believed to perceive the stimulation (five- alternative forced choice paradigm). Three blocks were performed per hand, where the stimulus duration was set to 1 s in the first and to 2 s in the last two blocks. Within each block, each finger was tested 5 times, with randomized order between fingers, resulting in 25 trials per block. In total, 15 trials per finger were recorded per hand.

##### Analysis

The average classification in % between truly stimulated and the estimated finger was determined across the three blocks per hand. In particular, the percentage of perceiving the correct finger (accuracy) was calculated for each finger. Also, the most likely perceived finger along with its classification likelihood was noted for each stimulated finger.

#### Psychophysics 2: MRI and Tactile Mapping via FMRI

##### MRI

A whole-brain anatomical MR image was acquired (3 T system MAGNETOM Prisma, Siemens Healthcare, Erlangen, Germany, 64-channel head coil: sagittal T1-weighted FLASH, repetition time = 9.5 ms, echo time = 4.23 ms, flip angle = 15°, acquisition matrix = 256 × 256, 176 partitions, resolution = 1 × 1 × 1 mm^3^, total acquisition time = 13 min). Also, three identical axial partial-volume fMRI data were obtained for mapping the fingertips of the affected hand in the contralateral Brodmann area (BA) 3b (gradient-echo echo-planar imaging, 2 × 2 × 4 mm^3^ resolution, 20 axial sections, repetition time = 2,000 ms, echo time = 36 ms, flip angle = 90°, field of view = 256 × 256 mm^2^, partial Fourier factor = 6/8), positioned such that the primary somatosensory cortices (SI) were covered.

##### Tactile Stimulation and fMRI Mapping

For tactile fingertip stimulation during fMRI, a piezo-electric stimulation device (QuaeroSys, St. Johann, Germany) with five 8-dot Braille display stimulation modules was used (Figure 2B), each positioned below one of the affected fingertips using our standardized protocol (32-Hz stimulation, for details see Schweisfurth et al., 2018). During the three fMRI runs, the five fingertips were repetitively and sequentially stimulated (stimulation and rest periods of 12 s length), resulting in a total measurement of 10:20 min per run. Data analysis was performed with BrainVoyager QX 2.6 (Goebel et al., 2006; Brain Innovation, Maastricht, The Netherlands), as described in more detail in (Schweisfurth et al., 2018). Functional runs were concatenated and co-registered to the anatomical image. Using standard general-linear-model analysis, the functional activations were explored and then projected onto the subject's contralateral cortical mesh. There, the vertices with the highest *t*-values were identified for each finger stimulation in SI.

### Results

#### Electrotactile Psychophysics

Using electrotactile fingertip stimulation, possible changes in sensitivity and possible mislocalizations between fingers were explored. The obtained sensation and discomfort thresholds are stated in Table 1. The thresholds for sensation were highly reproducible (with a difference of 5 ± 1 μs for the intact and 8 ± 13 μs for the impaired hand between the two repetitions). For the affected side, the sensation thresholds were consistently increased compared to the intact side, on average by a factor of 3. Similarly, the discomfort thresholds were increased by a factor of 1.6 for D5, 3 for D4 and even higher values (not assessible) for D1–D3 at the affected compared to the intact side.

**Table 1:** Results tactile psychophysics using electrostimulation.

		**D1**	**D2**	**D3**	**D4**	**D5**
PI (mA)	Both hands	8	8	10	8	8
PW_sen_ (μs)	Intact	35/35	50/50	30/35	35/35	35/35
	Affected	90/90	100/100	200/230	70/70	60/50
PW_def_ (μs)	Intact	55	110	45	60	55
	Affected	500	500	500	200	70
PW_unc_ (μs)	Intact	160	165	95	140	100
	Affected	>500	>500	>500	420	160
Accuracy (%)	Intact	100	100	100	87	100
	Affected	0	13	47	27	47

*For each hand and finger (D1–D5), the parameters determined and used during electrotactile stimulation are given. PI: used pulse intensity; PW_sen_, repetition $\frac{1}{2}$: minimal pulse width eliciting a sensation; PW_def_, pulse width used in mislocalization task; PW_unc_, pulse width eliciting uncomfortable sensation. Finally, the accuracy obtained during the mislocalization task is given*.

**Figure 3:**
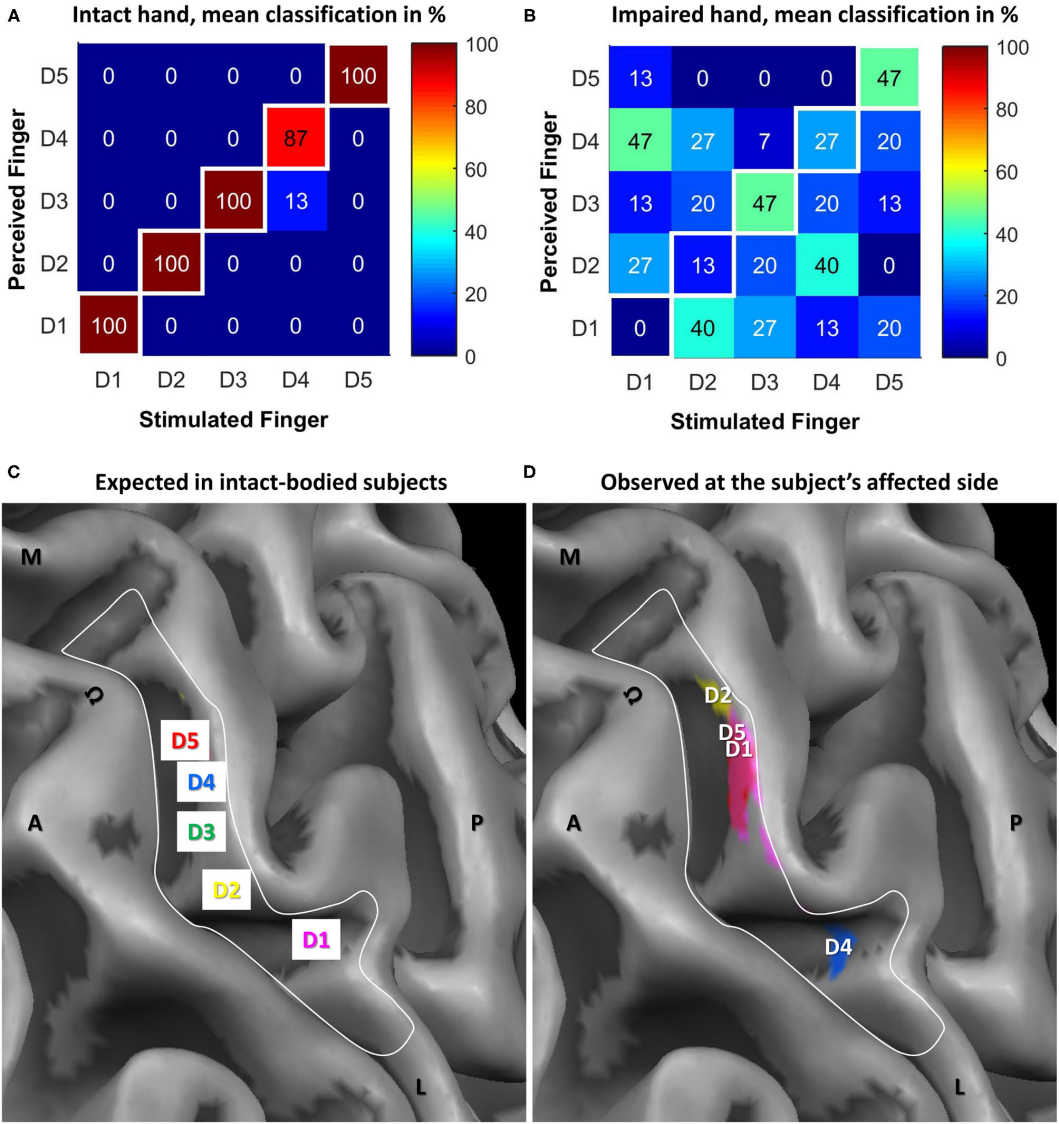
Results of the electrotactile stimulation and tactile mapping in S1 via fMRI as opposed to the expected cortical pattern in intact-bodied subjects. Confusion matrices for tactile psychophysics using electrostimulation for the **(A)** intact and **(B)** impaired hand. Shown are averaged results across the three blocks, percentage of how often the subject perceived one finger upon stimulation of the same (in white squares) or a different finger. The respective percentages are stated in numbers and further emphasized by color coding. The two lower panels show the hand representation in BA 3b expected in intact-bodied subjects and observed at the subject's affected side. In both panels, the same detail, covering the motor hand knob (Ω) and expected somatosensory hand area (white border) of the central sulcus of the subject's hemisphere contralateral to the affected side is shown. A, anterior' P, posterior; M, medial; L, lateral. **(C)** Schematic representation of the finger representation from lateral (D1) to medial (D5) as expected for an intact-bodied limb. **(D)** The subject's somatosensory representation of the affected fingers in assumed SI (cortical activations masked by that area). The most significant vertices are below the finger labels. Color code: Magenta = D1, yellow = D2, green = D3, blue = D4, red = D5.

During the mislocalization test, the subject presented with almost ideal performance (97 ± 6% correct responses) at the intact side (see Table 1; Figure 3A) whereas the perceived sensory map of the affected hand was disrupted (see Table 1; Figure 3B). The “best” performance with 47% correct responses was achieved for D3 and D5. For D4, the performance was slightly above chance (27% compared to 20% chance level), while for D2 (13%) and D1 (0%) performances lower than chance were observed. Upon stimulation of D1/D2/D4, the subject most commonly (between 40 and 47%) reported a sensation at D4/D1/D2.

#### Tactile Mapping in SI via fMRI

Tactile mapping of an intact arm typically results in a mediolateral fingertip succession from the little finger to the thumb in the contralateral hemisphere (see also Schweisfurth et al., 2011, 2014, 2018; Figure 3C). A very different map was found for the affected side (see Figure 3D). For all fingers except the middle finger (D3), significant activation was observed in the anatomically defined BA 3b in SI area. However, the succession and location of the representations was strongly altered from the expected pattern. D1 activation was found at expected D4; D2 activation was even further medial; reversely, D4 activation was rather at D1 representation. Only D5 was represented where it was expected (or slightly lateral to that). Hence, a mediolateral succession from D2 over D5 and D1 (almost co-located) to D4 was observed. The non-existence of D3 activation was consistent with the psychophysical observation of a very high sensation threshold.

## Discussion

While the full motor function of the elbow and wrist were recovered, the patient's hand remained paralyzed without any function in a stiff claw hand deformity, which is a common persisting deficit in BPI (Kim et al., 2003; Kandenwein et al., 2005; Chuang, 2010). Ulnar-nerve repair, dominantly restoring hand function, usually has worse outcome than median-nerve or radial-nerve repair, probably due to the relatively small fiber size and small volume of the innervated muscles (He et al., 2014). Our observation that stimulation of D1–D4 (median nerve area) almost never led to perception in the ulnar region (D5 perceived far below chance) along with that stimulation at D5 led to perception mainly at D5 indicate that a switch between ulnar and median nerve stump during surgery is highly unlikely. To the best of our knowledge, this is the first report on analyzing sensory recovery of fingertips in BPI. Except for D3, the patient was able to detect stimulation at each fingertip. However, the sensation thresholds of the affected side were increased compared to the intact side, on average being tripled. Consistently, the discomfort thresholds were also considerably higher at the affected compared to the intact side. Hence, the sensation was hyposensitive but available, showing that some reinnervation of the fingertips must have occurred. The overall sensory recovery of the hand is similar to comparable BPI cases followed by surgical reconstruction. Kandenwein et al. (2005) reported an impaired sensation of the hand in approximately 79% and an anesthesia of the hand for 10% of 134 analyzed BPI after surgery. For human neonates with reconstructed BPIs up to 30% recovery to a threshold perception of the affected hand not worse than two standard deviations below physiological mean value using two-point discrimination testing, meaning 70% of the neonates do not recover (Anand and Birch, 2002), is reported.

While a sensation was felt at all fingers (for D3 at the middle phalanx), the stimulation was mostly not perceived at the corresponding finger on the affected side. The percentage of correct responses in the mislocalization task ranged from 0% up to 47%. While the most commonly guessed finger was the correct one for D3 and D5, the subject most frequently (~45%) reported a sensation at D4/D1/D2 upon stimulation of D1/D2/D4, respectively. Interestingly, the first and last fingertip pairs are not even being innervated by the same nerve (median and ulnar nerve) in intact-bodied subjects excluding a confusion of the corresponding nerve fibers at the level of injury.

The cortical representations were altered compared to the assumed pre-accident map locations based on intact-subject observations (Schweisfurth et al., 2014, 2015). D3 representation was not identified probably due to a signal below the baseline noise level. Compared to the mapping of a control group, only D5 stimulation led to activation at the expected position. In contrast, D1 and D4 were represented in the usual D4 and D1 areas, respectively. The D2 representation was observed most medially, where the palm area is usually found. An explanation for the observed reorganization could be a complete denervation of D3 and a subsequent cortical reorganization with D4 moving laterally and D1 and D2 medially.

Another possible explanation would be that the peripheral nerve-fibers connecting the brain to D1 and D4 took wrong turns when regrowing through the median nerve repair site and ended up innervating the sensory receptors at the opposite locations instead. This would explain, why touching the fingertip of D1 leads to lighting up of the D4 area in the brain and vice versa. Consistently, touch of D1 mainly leads to perception of D4. This is of course in contradiction to what the patient sees. It is conceivable, that the ensuing conflict of information in the brain between touch and vision may have triggered plasticity processes making it possible, that the patient now is at least unsure, where to place the perception. This process may be a bit more advanced for D4, which is mapped to a D1 location but perceived between D2 and D4 with a slight preference for D2. In this line of thought, the mapping suggests that the fingertip of D2 may now be innervated by nerve fibers that originally ended in the palm, which is consistant with the fuzzy perception between D1 and D4 but clearly not D5, representing the area covered by the median nerve in the palm. This interpretation is consistent with data found in patients after macroreplantation of the arm showing similarly reduced (and partly disturbed) sensations in the replanted limb and comparable phenomena for cortical reorganization (Blume et al., 2014, 2018).

Dellon analyzed why the percentage of fair functional reconstruction of BPI remains low despite refinement in surgical techniques (Dellon, 1981, 1984). He attributed it to central re-education of the hand after peripheral nerve injury and repair. A peripheral reinnervation of the median-nerve skin territory was reported to be followed by reorganization of the cortex due to initial loss and subsequent regeneration of median nerve inputs to the brain. These reorganizational changes were specifically restricted to regions of the hand cortex where inputs from the median nerve were normally represented (Wall et al., 1986). Abnormal recovered tactile responsiveness from reinnervated skin regions was also observed, including abnormal locations or multiple cutaneous receptive fields. Even with microsurgical intrafascicular repair, Dellon stated, regenerating axons distally find some of their former “home” destroyed or degenerated. Other regenerating axons arrive distally to the correct local but wrong home receptors. These possibilities create the following potential alterations: a decreased number of normally functioning peripheral receptive fields, a new set of abnormal peripheral receptive fields (wrong fiber/receptor combinations, one fiber reinnervating multiple receptive fields, etc.) or dysesthetia (axons trapped in scar at repair site) (Dellon, 1981; Rosén et al., 2006). Our data seem to support this hypothesis.

The design of the study and the nature of a case report at a single time point limit the ability to generalize the results and the phenomena to other patients with brachial plexus palsy. Thus, it will be necessary to run and analyze the experiments in a larger cohort and at follow-ups and to compare to the results of routine sensory testing such as (static/dynamic) two-point-discrimination testing. Also, the observational nature of the case study obviously does not allow to draw causal inferences between the nerve injury and the observed sensory reorganization, as this would require additional criteria to be met (e.g., Gianicolo et al., 2020).

In sum, we report here the peripheral and central reorganization of mapping and perception of finger tip sensations in a case of high-level brachial plexus injury treated with a long-distance graft. We observed increased thresholds and a highly impaired discrimination/localization at the fingertip reinnervation sites together with only partial consistency between the altered perception (mislocalization test) and altered map (fMRI). These phenomena are probably occurring as reinnervation is followed by adaptive reactive activation of the cortex due to initial loss and subsequent regeneration of altered inputs to the brain. Our results demonstrate some somatosensory rehabilitation that is accompanied by a corresponding cortical reorganization in the primary somatosensory cortex. We interpret the ensuing discrepancy of central mapping and objectified subjective perception as neuroscientific evidence for a re-education of the brains model of the hand after peripheral nerve injury and repair.

## Data Availability Statement

The raw data supporting the conclusions of this article will be made available by the authors, without undue reservation.

## Ethics Statement

The studies involving human participants were reviewed and approved by Ethikkommission Universitätsmedizin Göttingen. The patients/participants provided their written informed consent to participate in this study. Written informed consent was obtained from the individual(s) for the publication of any potentially identifiable images or data included in this article.

## Author Contributions

JE had the idea, assisted the surgery, organized the rehabilitation, planned and assisted the measurements, and wrote the paper. MW planned and conducted the measurements, conducted the analysis, wrote the paper, and created the figures. AS and TW analyzed the data and revised the paper. JF helped in planning the fMRI measurements and revised the paper. NW assisted in analysis and revised the paper. DF helped in planning the psychophysics measurements and revised the paper GF conducted the surgery and revised the paper. All authors contributed to the article and approved the submitted version.

## Funding

The measurements were performed within the ERC grant DEMOVE (267888) (DF). The evaluation and interpretation was realized within the framework PROMPT FKZ: 13GW0340 A-F funded by the German Federal Ministry of Education and Research (JE, MW, and TW).

## Conflict of Interest

The authors declare that the research was conducted in the absence of any commercial or financial relationships that could be construed as a potential conflict of interest.

## Publisher's Note

All claims expressed in this article are solely those of the authors and do not necessarily represent those of their affiliated organizations, or those of the publisher, the editors and the reviewers. Any product that may be evaluated in this article, or claim that may be made by its manufacturer, is not guaranteed or endorsed by the publisher.
