# Supplementary figures and images for: Case Report: Plasticity in Central Sensory Finger Representation and Touch Perception After Microsurgical Reconstruction of Infraclavicular Brachial Plexus Injury
Source: Front Neurosci. 2022 Feb 25;16:793036. doi: 10.3389/fnins.2022.793036 (PMC8914191; doi:10.3389/fnins.2022.793036)

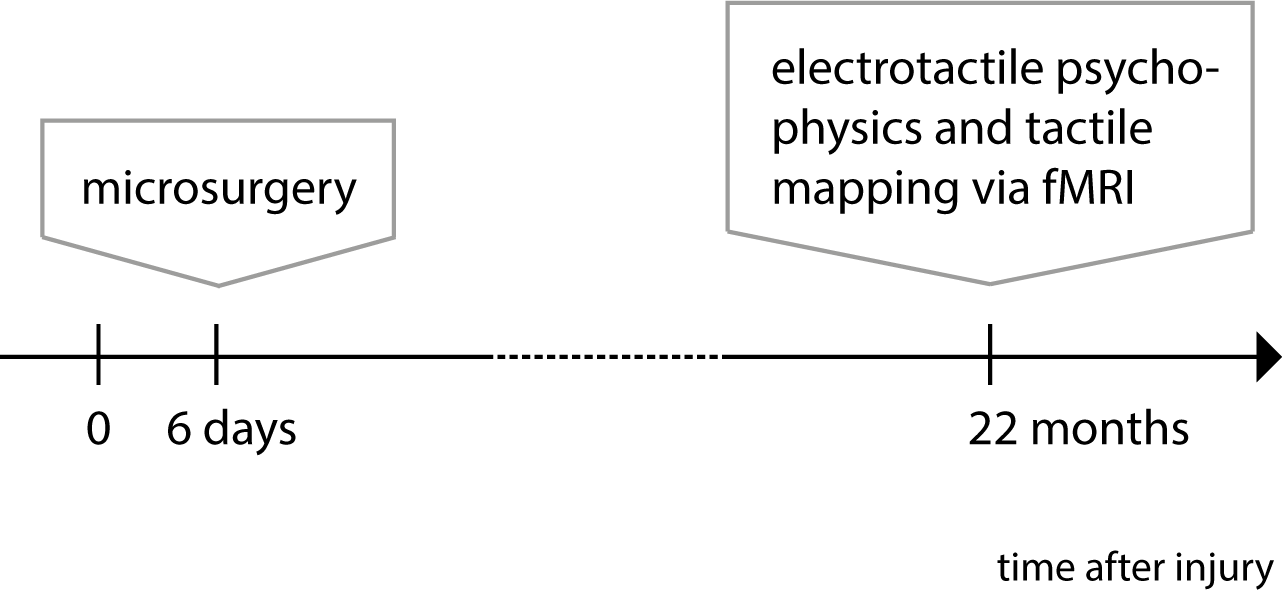

Supplement: Supplementary file 1 [file Image_1.TIF]
